# The approach of general practitioners in Lagos to the detection and evaluation of hypertension

**DOI:** 10.1186/s40885-015-0017-6

**Published:** 2015-06-17

**Authors:** Olagoke Korede Ale, Rotimi William Braimoh, Sunday O Olayemi

**Affiliations:** Cardiology Unit, Department of Medicine, University of Lagos/Lagos University Teaching Hospital, Lagos, Nigeria; Nephrology Unit, Department of Medicine, College of Medicine, University of Lagos, Lagos, Nigeria

**Keywords:** Hypertension, Detection, Evaluation, General medical practitioners, Hypertension guidelines

## Abstract

**Introduction:**

Hypertension (HTN) control is a major public health and clinical challenge. A number of guidelines exist globally to assist in tackling this challenge. The aim of this study was to determine conformity of the HTN detection and evaluation practices of a sample of Lagos-based general practitioners (GPs) to international guidelines.

**Methods:**

Self-administered structured questionnaires were used to collect data from a cohort of GPs attending continuing medical education programs in Lagos.

**Results:**

Out of the 460 GPs that were approached, 435 agreed to participate in the study, with questionnaires from 403 GPs analyzed. The average age and number of years post-registration of the GPs were 40.0 ± 11.3 years and 14.3 ± 11.1 years, respectively. Two thirds (*n* = 269) were in private practice. Their daily average total and HTN patients’ loads were 17.4 ± 14.3 and 4.4 ± 3.5, respectively. Awareness of HTN guidelines was 46.7% (*n* = 188), while 18.1% (*n* = 73) was able to name one or more HTN guidelines. The approaches of these GPs to the detection and evaluation of HTN and their relationships to the GPs’ experience were heterogeneous.

**Discussion:**

The approach of the GPs to detection and evaluation of HTN though heterogeneous is unsatisfactory and may partly contribute to poor HTN control in Nigeria. Strengthening the capacity of GPs in this regard through continuous medical education may greatly improve HTN control.

## Introduction

Hypertension (HTN) is the leading cardiovascular (CV) risk factor for morbidity and mortality worldwide [1]. It is also the most common condition in primary care [2]. Two thirds of an estimated 26.4% of the global adult population having HTN live in developing countries, with West Africa having HTN prevalence of 19.3%–54.6% [3,4].

Undetected, belatedly detected, untreated, or inappropriately treated HTN is associated with increased risk of CV morbidity and mortality, while good HTN management is associated with good CV prognosis [2,5,6]. Numerous data from HTN studies and clinical trials have provided newfound capabilities for lowering blood pressure (BP) in almost every person with HTN [6]. These data collated globally have been summarized into HTN guidelines.

The International Forum for Hypertension Control and Prevention in Africa (IFHA) recommendations for prevention, diagnosis, and management of HTN and CV risk factors in sub-Saharan Africa (SSA) is one of the numerous HTN guidelines available globally [7]. The IFHA guidelines follow the main lines stated in the 2003 WHO/ISH Statement on Management of HTN [8], the 2002 WHO CV Risk Management Package in Low- and Medium-Resource Settings [9], the American JNC 7 guidelines [10], the European guidelines [11], a consensus statement of the HTN in African-American working group of the International Society on HTN in Blacks [12], and European Society of HTN recommendations for blood pressure measurement [7,13].

Despite the above, HTN still remains a major public health concern with increasing prevalence and poor control worldwide [4,6,8,14,15]. HTN studies in Nigeria and Ghana showed 14%–73% awareness, 3%–86% on treatment, and 2%–13% with controlled BP [4]. This global problem of poor HTN control is partly due to a failure to ensure that people who have HTN are aware of it [15]. Therefore, the implementation of appropriate mechanisms required for the detection of HTN which are included in guidelines should positively impact hypertension control [7,8,10-12].

HTN often coexists with other CV risk factors, such as diabetes, dyslipidemia, tobacco use, and obesity, which are often inadequately addressed [8]. Hence, guidelines recommend total CV risk assessment of patients because it allows for a better adaptation of a patient’s clinical management [7,8,10]. CV risk assessment is done through medical history, physical examination, laboratory tests, and other diagnostic procedures.

A very high prevalence of target organ damage in Nigerians newly diagnosed with HTN suggests delayed detection of HTN [16]. This makes proper evaluation of newly diagnosed hypertensive persons imperative as recommended by HTN guidelines [7,8,10-12,17]. HTN has been reported to be poorly diagnosed in general medical practice [11]. Physician surveys and practice audits have revealed that the approach to the diagnosis, investigation, and treatment of HTN differs among geographical areas and over time [18]. The approach of primary care physicians may among other reasons such as the asymptomatic nature of HTN be partly responsible for poor detection and control of HTN. This study sought to determine the approach of general medical practitioners (GPs) in Lagos, the commercial capital and former administrative capital of Nigeria to the detection and evaluation of high BP and its agreement with the IFHA guidelines. We also determined the relationships of the GPs’ experience to their approach.

## Methods

The study was an exploratory survey of Lagos-based GPs attending continuing medical education program in Lagos, Nigeria. All the attending GPs who met the criteria for the study were approached. Inclusion criterion was full registration with the Medical and Dental Council of Nigeria. Physicians with specialty training in internal medicine or any of its subspecialties were excluded.

Ethical clearance was obtained from the Ethics and Research Committee of the Lagos University Teaching Hospital. The study was conducted to conform to the ethical tenets developed by the World Medical Association, as espoused in the Declaration of Helsinki. Permission to conduct the survey was obtained from the program organizers. Verbal consent of each participant was obtained.

### Survey instrument

Anonymous self-administered questionnaire developed in English consisting of open- and closed-ended questions was used. The closed-ended questions had either yes/no or Likert-type scale responses.

The study questionnaire had three basic themes as follows: (1) type of practice—private versus government and number of patients seen, (2) detection of HTN—frequency of BP checks in patients, rest before BP measurement, number of BP readings, and BP threshold levels; and (3) evaluation: (3a) clinical (personal history of diabetes mellitus, alcohol and tobacco habits, family history of diabetes and HTN, evaluation for obesity, and BP measurement in both arms on first visit) and (3b) laboratory/ancillary (urinalysis, serum electrolytes and creatinine, blood glucose, lipogram, electrocardiogram, and fundoscopy) evaluation of newly diagnosed hypertensive patients. A yes or no question on awareness of HTN guidelines was included, and physicians with affirmative responses were asked to list one or more HTN guidelines.

### Statistical analysis

#### Outcome variables

Definitions were adopted for binary outcomes based on the IFHA recommendations for prevention diagnosis and management of HTN and cardiovascular risk factors in sub-Saharan Africa [7].

Likert-type scale responses were transformed into dichotomous responses of appropriate/yes (“always done” and “often or usually done”) and inappropriate/no (“sometimes done,” “occasionally done,” “rarely or never done”) practice.

Another Likert-like scale (strongly agree, agree, neutral/undecided, disagree, and strongly disagree) response to the statement “uncomplicated hypertension is usually asymptomatic” was transformed into yes (strongly agree, agree) and no (neutral/undecided, disagree, and strongly disagree).

### Statistics

The data were analyzed using Statistical Package for the Social Sciences (SPSS, version 17.0). Descriptive statistics were used to report the findings. Categorical and continuous variables were expressed as proportions and means ± SD, respectively. Student *t*-test or Pearson chi-square test was used when appropriate. All tests were two-sided, and values were considered statistically significant if *p* < 0.05. Cross tables were done between demography of respondents and questions.

## Results

Out of the 460 physicians that were contacted, 435 agreed to participate with 403 of the questionnaires found suitable for analysis. The demographic characteristics of the respondents by experience (number of years post-registration) are shown in Table 1. The mean age and duration of practice of the GPs are 40.00 ± 11.34 years and 14.3 ± 11.1 years, respectively. Most of the GPs were males (61.8%, *n* = 249), in private practice (66.7%, *n* = 269) and unaware of HTN guidelines (53.3%, *n* = 215).

**Table 1:** **Demography of the respondents according to the number of years post registration**

**Variable (** ***n*** **)**	**All**	**Years post-registration**		
	***n*** **(%)/mean ± SD**	**<10 years**	**≥10 yrs**	***χ*** ^**2**^ **/** ***p*** **value**
		***n*** **(%)/mean ± SD**	***n*** **(%)/mean ± SD**	
Number of physicians	403 (100%)	189 (48.9%)	214 (53.1%)	3.10/0.078
Age (397)	40.00 **±** 11.34	30.28 **±** 2.64	48.45 **±** 8.96	<0.001
Sex (403)				
Male	249 (61.8%)	95 (51.6%)	154 (72%)	20.01/<0.001
Female	154 (38.2%)	94 (48.4%)	60 (28%)	
Years post-registration (403)	14.30 ± 11.10	4.12 ± 1.69	23.29 ± 7.55	<0.001
Type of practice (403)				
Private	269 (66.7%)	99 (52.3%)	170(79.4%)	33.11/<0.001
Government	134 (33.3%)	90 (47.7%)	44 (20.6%)	
Number of patients seen per day (403)	17.39 ± 14.25	17.24 ± 16.42	18.46 ± 12.04	0.39
Number of HTN patients seen per day (396)	4.38 ± 3.50	5.30 ± 3.90	3.57 ± 2. 37	<0.001
Aware of guidelines (403)	188 (46.7%)	88 (46.6%)	100 (46.7%)	0.001/0.97
Able to name guidelines (403)	73 (18.1%)	53 (28%)	20 (9.3%)	23.65/<0.001

HTN hypertension.

Table 2 shows the approach of the GPs to the detection of HTN. A quarter (25.3%, *n* = 102) of the GPs used incorrect BP threshold for the diagnosis of HTN. Eighty eight (86.3%) and 14 (13.7%) of these GPs gave threshold values higher and lower respectively than the correct BP threshold of ≥140/90 mmHg. Apart from threshold for HTN diagnosis and routine BP check, there were no significant differences in the approaches of the older and recent medical graduates to the detection of HTN.

**Table 2:** **Detection of hypertension by the respondents according to the number of years post registration**

**Variable (** ***n*** **)**	**All**	**Years post-registration**		
	***n*** **(%)**	**<10 years**	**≥10 years**	***χ*** ^**2**^ **/** ***p*** **value**
		***n*** **(%)**	***n*** **(%)**	
Correct BP threshold for HTN diagnosis (403)	301 (74.7%)	151 (79.9%)	150 (70%)	5.10/0.024
Routinely check BP in practice (392)	273 (69.6%)	87 (48.9%)	186 (86.9%)	66.51/<0.001
Allow short rest before measuring BP (390)	103 (26.4%)	45 (24.8%)	58 (27.8%)	0.42/0.52
Take ≥2 BP readings before diagnosing HTN (403)	398 (98.8%)	189 (100%)	209 (97.7%)	4.47/0.034
Measure BP in both arms during first visit (390)	63 (16.2%)	23 (12.8%)	40 (18.6%)	2.67/0.10
Agree that uncomplicated HTN is asymptomatic (403)	281 (69.7%)	124 (65.6%)	157 (73.4%)	2.86/0.09

*BP* blood pressure, *HTN* hypertension.

Table 3 shows the responses of the GPs to the questions on the evaluation of HTN. Apart from evaluation for obesity (45.8%, *n* = 183) and dyslipidemia (41.2%, *n* = 166), majority of the GPs routinely carried out clinical and laboratory evaluation on their newly diagnosed hypertensive patients. The recently qualified GPs were better than the older ones in terms of personal history of diabetes, history of alcohol intake, and serum electrolytes and creatinine assay (*p* < 0.05). The converse is the case urinalysis and physical activity evaluation (*p* < 0.05).

**Table 3:** **Clinical and laboratory evaluation by the respondents according to the number of years post-registration**

**Evaluation (** ***n*** **)**	**All**	**Years post-registration**		
	***n*** **(%)**	**<10 years**	**≥10 years**	***χ*** ^**2**^ **/** ***p*** **value**
		***n*** **(%)**	***n*** **(%)**	
FH of HTN (398)	349 (87.7%)	155 (84.2%)	194 (90.7%)	3.77/0.052
FH of DM (403)	305 (75.7%)	137 (72.5%)	168 (78.5%)	1.98/0.16
PH of DM (400)	312 (78%)	158 (84.9%)	154 (72%)	9.78/0.002
Obesity evaluation (400)	183 (45.8%)	83 (43.9%)	100 (47.4%)	0.49/0.49
Alcohol history (403)	297 (73.7%)	148 (78.3%)	149 (69.6%)	3.901/0.048
Tobacco history (398)	297 (74.6%)	133 (70.4%)	164 (78.5%)	3.44/0.064
Physical activity (383)	251 (65.5%)	109 (59.2%)	142 (71.4%)	6.22/0.013
Urinalysis (403)	324 (80.4%)	129 (68.3%)	196 (91.6%)	33.3/<0.001
Blood glucose (398)	248 (62.3%)	117 (61.9%)	131 (62.7%)	0.028/0.87
E U Cr (399)	245 (61.4%)	131 (69.3%)	114 (54.3%)	9.48/0.002
Lipogram (403)	166 (41.2%)	82 (43.4%)	84 (43.4%)	0.71/0.40
Fundoscopy (400)	21 (5.25%)	10 (5.3%)	11 (5.2%)	0.001/0.97
Electrocardiography (398)	204 (51.3%)	105 (55.6%)	99 (47.4%)	2.67/0.103

*FH* family history, *HTN* hypertension, *PH* personal history, *DM* diabetes mellitus, *E U Cr* serum electrolytes and creatinine.

Table 4 shows the relationships of the total number of patients seen per day, the number of hypertensive patients seen per day and the number of years post-registration of respondents to selected knowledge and practices of the physicians.

**Table 4:** **Relationships of the number of patients seen per day, age and experience of physicians to selected practices**

**Practice**		**Total number of patients seen per day**	***p*** **value**	**Number of HTN patients seen per day**	***p*** **value**	**Age of physicians (years)**	***p*** **value**	**Years post-registration (years)**	***p*** **value**
		**Mean ± SD**		**Mean ± SD**		**Mean ± SD**		**Mean ± SD**	
Routinely check BP	Y	17.7 ± 11.8	0.33	4.2 ± 3.2	0.34	43.8 ± 11. 5	<0.01	17.8 ± 11.2	<0.01
	N	19.2 ± 19.9		4.6 ± 3.9		32.1 ± 5.5		7.1 ± 6.3	
Correct HTN threshold	Y	13.2 ± 14.5	0.53	3.8 ± 0.9	0.05	38.9 ± 10.6	<0.01	13.4 ± 10.8	<0.01
	N	17.1 ± 13.5		4.6 ± 3.7		43.3 ± 12.7		17.0 ± 11.7	
PH of diabetes	Y	13.2 ± 15.4	0.58	4.6 ± 3.8	0.02	39.5 ± 10.6	0.03	13.4 ± 10.8	<0.01
	N	17.2 ± 9.1		3.6 ± 2.2		42.6 ± 13.3		17.9 ± 11.5	
FH of diabetes	Y	16.5 ± 10.9	<0.01	4.4 ± 3.8	0.81	40.3 ± 10.6	0.57	14.7 ± 10.7	0.24
	N	22.1 ± 1.1		4.3 ± 0.5		39.5 ± 13.5		13.2 ± 12.3	
FH of HTN	Y	16.4 ± 10.5	<0.01	4.4 ± 3.6	0.76	40.5 ± 10.8	0.25	14.8 ± 10.8	0.11
	N	28.3 ± 27.7		4.2 ± 2.7		38.4 ± 15.0		12.0 ± 13.0	
Obesity assessment	Y	16.6 ± 0.8	0.11	4.2 ± 3.8	0.47	40.5 ± 10.4	0.57	14.2 ± 10.8	0.86
	N	13.9 ± 16.7		4.5 ± 3.8		39.8 ± 12.2		14.4 ± 10.5	
BG assessment	Y	18.0 ± 11.7	0.80	4.6 ± 3.8	0.02	40.1 ± 11.6	0.61	15.6 ± 11.1	<0.01
	N	17.6 ± 17.9		3.8 ± 2.9		39.5 ± 10.4		8.9 ± 9.5	
Urinalysis	Y	16.8 ± 11.3	<0.01	4.0 ± 3.5	<0.01	41.3 ± 11.5	<0.01	14.1 ± 11.2	0.84
	N	22.2 ± 22.3		6.0 ± 2.9		35.1 ± 9.2		14.3 ± 10.9	
E U Cr	Y	17.6 ± 11.3	0.47	4.6 ± 3.8	0.14	39.1 ± 11.2	0.047	13.0 ± 10.9	<0.01
	N	18.6 ± 18.1		4.1 ± 2.9		41.4 ± 11.4		16.0 ± 11.0	
ECG	Y	18.2 ± 12.1	0.72	4.7 ± 3.7	0.14	38.9 ± 10.3	0.15	12.9 ± 10.3	0.04
	N	17.7 ± 16.4		4.1 ± 3.3		40.5 ± 11.3		15.2 ± 11.3	
Lipogram	Y	18.0 ± 12.9	0.93	5.1 ± 4.0	<0.01	39.6 ± 10.6	0.52	13.6 ± 11.2	0.4
	N	17.8 ± 15.2		3.9 ± .07		40.4 ± 11.9		14.7 ± 11.1	

*HTN* hypertension, *FH* family history, *PH* personal history, *DM* diabetes mellitus, *BG* blood glucose, *E U Cr* serum electrolytes and creatinine. *ECG* electrocardiography, *Y* yes, *N* no.

## Discussion

Hypertension is largely asymptomatic in nature and can only be diagnosed by measuring the BP. This informed IFHA guideline recommendation that health-care professionals should measure the BP at every encounter with health-care seekers [7]. A large proportion (30.4%) of the respondents in this study did not routinely check the BP of their adult patients in consultation, with this practice commoner among the younger graduates. This is less than 52.4% reported by Olubodun in a study of 42 Nigerian GPs [19]. A practice audit of 400 Italian GPs by Filippi et al. showed that 27.8% of the patients seen over a period of 1 year had no recorded BP values [20]. Noubiap et al. in study of 77 GPs based in Cameroon reported that 80.5% of them routinely check the BP of their adult patients in consultation [21]. Data on 2,618 consecutive adult patients presenting to 99 Australian GPs over a 5-week period showed that BP had not been recorded for 13% of the patients [22]. However; a survey of 100 GPs in Lagos by Ajuluchukwu et al. reported that all the respondents measured the BP of their adult clients most of the time [23]. In addition to differences in methodology and varying abilities of GPs to recall, the wide variability observed globally in the proportions of GPs routinely checking the BP of adults in consultation may be due to differences in patient load and the perceptions of these GPs on the enormity of the public health challenge posed by HTN.

A disproportionately higher number of the more experienced physicians were in private practice when compared with the less experienced ones in our study. A more meticulous approach to patient care coupled with lighter patient load in private practice when compared with government practice may explain the better practice in terms of routine BP checks observed among the more experienced GPs. The practice of not routinely checking BP may account for the high prevalence of undetected HBP and a consequent high prevalence of target organ damage reported in newly diagnosed hypertensive subjects in Nigeria [4,16].

The BP threshold for the diagnosis of hypertension is the BP at which the benefits of drug treatment have been definitely established in randomized placebo controlled trials. This threshold has been a moving target which had witnessed reductions over the past years as more data on HTN streamed in. The majority (74.7%) of the respondents used correct BP threshold for diagnosing HTN. This proportion is higher than the 63.6% and 69.4% reported for Cameroon- and Pakistan-based GPs, respectively [21,24]. A large majority (86%) of the GPs using incorrect BP threshold values used values higher than the correct BP threshold with this being commoner among the more experienced GPs. This not only implies under diagnosis of HTN but also suggests that the more experienced GPs may be less familiar with the recent hypertension guidelines having lower BP threshold for the diagnosis of HTN.

Overweight/obesity is not just a highly prevalent condition in West Africa with a prevalence of 4%–49% in Nigeria; its prevalence is trending upwards in the region [4,25]. HTN prevalence across various populations increase with average body mass index, and it is known that 75% of the incidence of HTN is related directly to obesity [26,27]. Less than half of the respondents in the current study routinely evaluated their newly diagnosed hypertensive clients for obesity, thereby undermining the effect of obesity on hypertension especially among the Black. Indeed, obesity has been found to be the most important cause of uncontrolled BP in certain populations and is also associated with increased morbidity and mortality [28].

Lipogram was routinely carried out by 41.2% of the GPs in the current study. This is in contrast to higher figures reported for Indian (82.5%) and Cameroonian (50.7%) GPs [21,29]. This may be due to the suggestion by the IFHA guidelines that lipid studies may be unimportant in the evaluation of newly diagnosed hypertensive Blacks of SSA [7]. This was premised on suggestions by the older publications that serum lipid levels in Blacks of SSA are low [30]. This practice is rather worrisome as current data show that lipid abnormalities are not only common in Nigerians newly presenting HTN, but it also worsens with severity of HTN [31 32]. These publications further recommended screening for lipid abnormalities as an important investigation in the evaluation of Nigerians presenting with HTN [31,32]. It is well known that institution of therapy for dyslipidemia when detected is desirable to reduce the adverse multiplicative effects of coexisting HTN and lipid abnormalities [33].

Tobacco causes an estimated 10% of CVD and is the second leading cause of CVD after HTN with both its chewing and smoking causing CVD [34]. Majority of the respondents inquired about tobacco use showing that they understood its implications as a comorbid factor in HTN management.

The presence of cardiac damage in two thirds of the newly diagnosed hypertensive Nigerians makes electrocardiography (ECG) imperative in their management [16]. ECG was routinely carried out by only half (51.3%) of the GPs in this study. This is comparable to 42.7% reported for GPs in Cameroon which is also a sub-Saharan African country, but far lower than 94.8% reported for GPs in India [21,29]. Ignorance of the magnitude of hypertensive heart disease among Nigerians by this cohort may underlie this practice, since facilities for electrocardiography are readily and widely available in Lagos. Affordability to Nigerian patients of this evaluation tool may also be a reason for both its low utilization by GPs in our study and the disparity observed between Nigerian and Indian GPs in the usage of electrocardiography.

Retinopathy has been reported to occur in 71% of Nigerians newly diagnosed with HTN [16]. However, examination of the optic fundi was rarely (5.25%) done by GPs in our study. This is lower than 10.4% reported Noubiap et al. for GPs in Cameroon [21]. This very poor utilization of fundoscopy by the GPs in these studies may be due to limited expertise and/or time constraints. The importance of fundoscopy in the management and prognostication of hypertensive patients may also not be adequately appreciated by these physicians.

This study has some limitations. First, the use of self-administered questionnaire is influenced by recall and the tendency to state the ideal rather than the actual practice; hence, a study based on medical database would have been ideal. In addition, though the participation was voluntary, the use of a cohort of GPs attending continuing medical education program may not rule out selection bias. In spite of these limitations, this study provides a benchmark for further studies in this area.

## Conclusion

Compliance to guidelines on various aspects of the detection and evaluation of hypertension by this cohort though heterogeneous is unsatisfactory. This engenders the need for continuous education on hypertension for GPs in Lagos in order to be kept abreast with current management strategies on hypertension.

## Abbreviations

HTN: Hypertension
GPs: General practitioners
BP: Blood pressure
CV: Cardiovascular
SSA: Sub-Saharan Africa
IFHA: International Forum for Hypertension Control and Prevention in Africa
WHO: World Health Organization
ISH: International Society of Hypertension
JNC7: The Seventh Report of the Joint National Committee on Prevention, Detection, Evaluation, and Treatment of High Blood Pressure
